# Chromatin deregulation in disease

**DOI:** 10.1007/s00412-015-0530-0

**Published:** 2015-07-19

**Authors:** Anne C. Mirabella, Benjamin M. Foster, Till Bartke

**Affiliations:** Chromatin Biochemistry Group, MRC Clinical Sciences Centre, Imperial College London, Du Cane Road, London, W12 0NN UK

**Keywords:** Gene expression, Epigenetics, Chromatin, DNA methylation, Histone modifications, Chromatin remodeling

## Abstract

The regulation of chromatin by epigenetic mechanisms plays a central role in gene expression and is essential for development and maintenance of cell identity and function. Aberrant chromatin regulation is observed in many diseases where it leads to defects in epigenetic gene regulation resulting in pathological gene expression programmes. These defects are caused by inherited or acquired mutations in genes encoding enzymes that deposit or remove DNA and histone modifications and that shape chromatin architecture. Chromatin deregulation often results in neurodevelopmental disorders and intellectual disabilities, frequently linked to physical and developmental abnormalities, but can also cause neurodegenerative diseases, immunodeficiency, or muscle wasting syndromes. Epigenetic diseases can either be of monogenic origin or manifest themselves as complex multifactorial diseases such as in congenital heart disease, autism spectrum disorders, or cancer in which mutations in chromatin regulators are contributing factors. The environment directly influences the epigenome and can induce changes that cause or predispose to diseases through risk factors such as stress, malnutrition or exposure to harmful chemicals. The plasticity of chromatin regulation makes targeting the enzymatic machinery an attractive strategy for therapeutic intervention and an increasing number of small molecule inhibitors against a variety of epigenetic regulators are in clinical use or under development. In this review, we will give an overview of the molecular lesions that underlie epigenetic diseases, and we will discuss the impact of the environment and prospects for epigenetic therapies.

## Introduction

Epigenetics describes biological phenomena resulting in heritable changes to gene expression that occur without alterations to the DNA sequence. Nearly all cells in the human body have the same genotype; however, during development they acquire a multitude of different phenotypes, resulting from differences in their gene expression patterns. Selective gene expression from the genome is shaped by epigenetic mechanisms and as a consequence cells vary from tissue to tissue in their epigenomes whilst the genome remains largely unchanged.

**Fig. 1:**
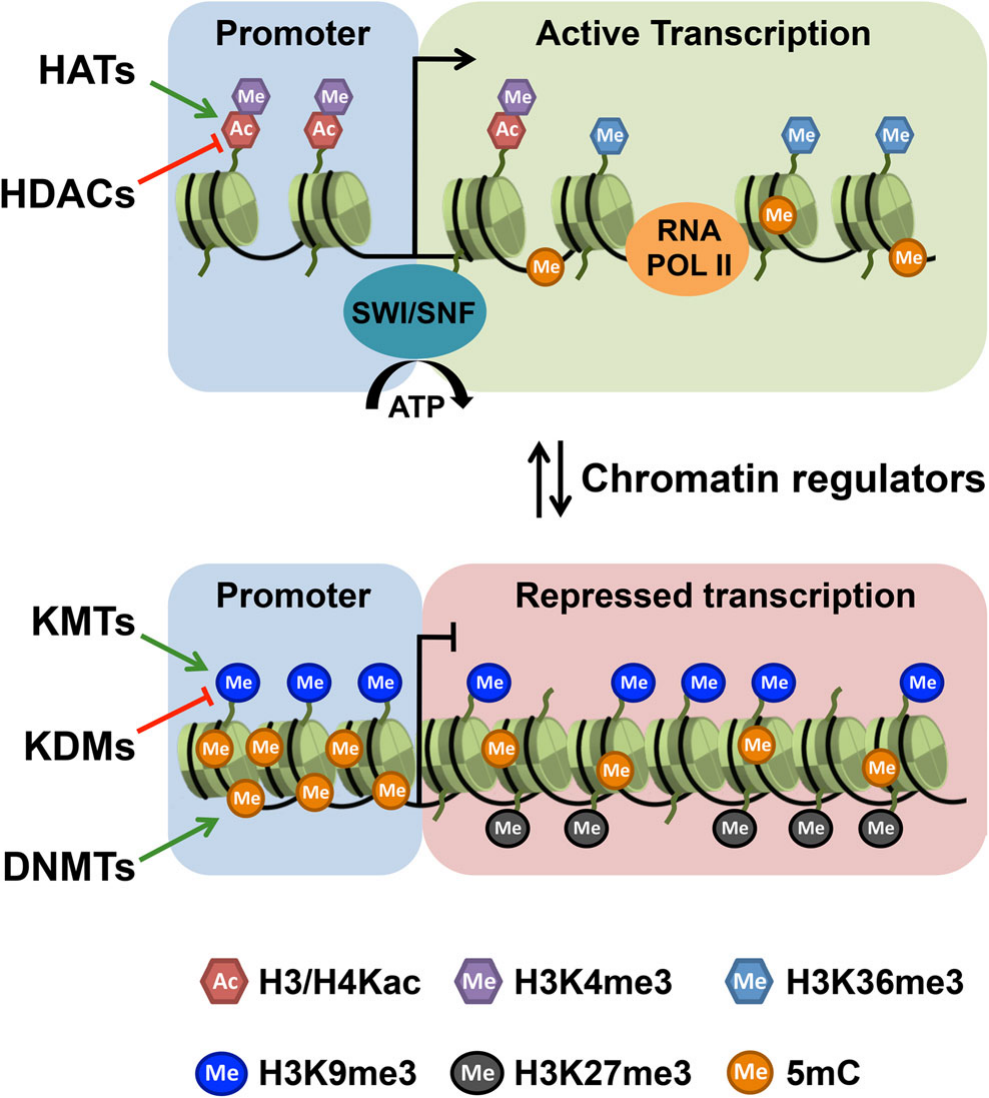
Mechanisms of chromatin regulation. Actively transcribed genes are found in open euchromatin and are associated with histone acetylation (*H3/H4Kac*) and tri-methylation of H3 lysine 4 (*H3K4me3*) at promotors, and tri-methylation of H3 lysine 36 (*H3K36me3*) over the gene body. Nucleosome positioning at promoters is regulated by ATP-dependent chromatin remodelers (*SWI/SNF*). Silenced genes are associated with densely packed heterochromatin marked by DNA methylation (*5mC*) and H3 lysine 9 tri-methylation (*H3K9me3*) or in silenced polycomb domains marked by tri-methylation of H3 lysine 27 (*H3K27me3*). Chromatin modifications are deposited by chromatin modifying enzymes such as DNA methyltransferases (*DNMTs*), histone acetyl transferases (*HATs*) or histone methyl transferases (*KMTs*) and removed by de-modifying enzymes such as histone deacetylases (*HDACs*) or histone demethylases (*KDMs*). These enzymes constitute intervention points for epigenetic therapies. A list of inhibitors against chromatin regulators is provided in Table 2

In eukaryotes, the majority of the DNA resides in the nucleus where it is packaged into a highly condensed structure called chromatin. The primary and repeating units of chromatin are the nucleosomes, which consist of 147 bp of DNA wrapped around an octamer of histone proteins formed by two copies of each of the core histones H2A, H2B, H3 and H4, with the linker histone H1 bound to the DNA between nucleosomes (reviewed in Luger et al. 2012). Chromatin is dynamically and tightly regulated and many factors and mechanisms participate in this process (Fig. 1). These include the covalent modification of DNA and histone proteins, regulation of nucleosome assembly by histone chaperones, ATP-dependent remodeling of nucleosomes by chromatin remodeling factors to control DNA accessibility, and exchange of canonical histones against histone variants. These processes allow epigenetic information to be encoded in chromatin and together they act in regulating gene expression, DNA repair and replication.

The compaction of the genetic material into chromatin generally leads to repression of transcription, and genes and their regulatory regions need to be made accessible to transcription factors and chromatin associated proteins in order for gene expression to occur (Pazin et al. 1994). The central importance of epigenetic mechanisms in establishing the correct gene expression programmes also means that pathological states may result if epigenetic regulation is compromised and the role of epigenetics in many diseases is now widely appreciated. The reversible nature of the underlying mechanisms, however, also provides plausible prevention and treatment options for diseases with epigenetic origins.

**Fig. 2:**
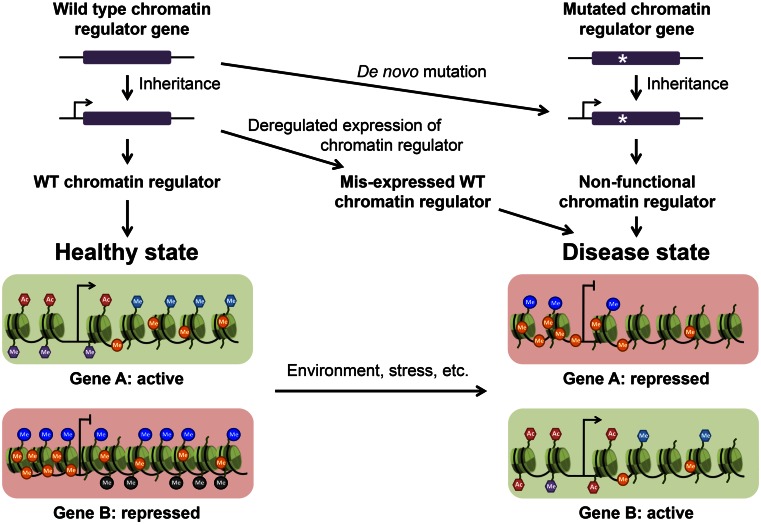
Chromatin deregulation in disease. Pathologies can result from changes in gene expression programmes caused by aberrant DNA methylation and histone modification patterns. These changes can be caused by environmental stresses that affect the chromatin state, deregulated expression of wild type (*WT*) chromatin regulators, or as a result of mutations (either inherited or acquired de novo) in genes encoding chromatin regulatory proteins

In this review, we will give an overview of the molecular lesions that can cause epigenetic diseases. These diseases are typically consequences of mutations in epigenetic regulators that are either inherited or acquired de novo, or originate from exposure to harmful environments (Fig. 2). We will focus on mechanisms directly related to chromatin regulation and we will discuss how diseases can arise when this regulation goes awry. We will concentrate on the three main mechanisms that govern chromatin regulation: DNA modification, histone post-translational modifications (PTMs) and chromatin remodeling, all of which play major roles in gene regulation and are implicated in the formation of a multitude of epigenetic diseases (Table 1). A further important mechanism in controlling chromatin function is the exchange of canonical histones against histone variants. As there is little information concerning the role of histone variants in diseases beyond cancer, we will not further discuss them here; instead, we refer the reader to recent reviews summarizing our current knowledge for the field of cancer (Cantarino et al. 2013; Vardabasso et al. 2014).

**Table 1:** Diseases associated with chromatin deregulation

Disease	Affected Gene	Mechanism	Symptoms	OMIM reference^a^	References
DNA methylation					
Parkinson’s	TNFA (TNF-alpha); SNCA (alpha-synuclein)	Hypo-methylation at promoter regions	Muscular rigidity, tremors	168600163890615869	See review by Lu et al. (2013)
Alzheimer’s	many	Locus-specific hypo- or hyper methylation	Dementia and gradually worsening ability to form new memories	104300	
Huntington’s	SCA7 locus; A2aR	DNA methylation at CTCF binding sites (CAG repeats) at SCA7 locus, increased 5mC at A2aR promoter	Chorea, rigidity and dementia	143100	
Fragile X syndrome	FMR1	Hyper-methylation at CGG repeats repressing FMR1 expression	Moderate to severe mental retardation, macroorchidism, and distinct facial features, including long face, large ears, and prominent jaw	300624	Jin and Warren (2000); Sutcliffe et al. (1992); Wang and Griffith (1996)
Facioscapulohumeral muscular dystrophy (FSHD)	FSHD locus 4q35	Reduced DNA CpG methylation due to a reduction in number of D4Z4 repeats	Progressive wasting of facial, upper arm and shoulder girdle muscles	158900	Cabianca et al. (2012)
Prader-Willi syndrome	15q11-q13 locus	Imprinting	Infantile hypotonia, obesity due to hyperphagia and mental retardation	176270	Cassidy and Schwartz (1998)
Angelman syndrome	UBE3A (E6AP)	Imprinting	Severe mental retardation	105830	Cassidy and Schwartz (1998); Kishino et al. (1997)
Rett syndrome	MECP2	Reduced Methyl-DNA binding due to mutation in *MECP2*	Mental retardation involving loss of acquired speech	312750	Amir et al. (1999); Bird (2008)
Immunodeficiency, centromeric region instability, and facial anomalies syndrome (ICF)	DNMT3B	Rearrangements and reduced CpG DNA methylation at satellite repeat regions on Chr 1, 9 and 16 due to mutation in DNMT3B	Facial dysmorphism, immunoglobulin deficiency	242860	Ehrlich et al. (2008); Hansen et al. (1999); Xie et al. (2006); Xu et al. (1999)
Histone modifications					
Rubinstein–Taybi syndrome	CREBBP (CBP, KAT3A); EP300 (KAT3B)	Reduced HAT activity due to mutations in *CBP* and *EP300*	Mental retardation, postnatal growth deficiency, microcephaly, broad thumbs and halluces, and dysmorphic facial features	180849 613684	Petrij et al. (1995); Roelfsema et al. (2005)
Amyotrophic lateral sclerosis (ALS)	FUS (TLS)	Reduced HAT activity due to deregulation via FUS	Neurodegenerative disorder characterised by the death of motor neurons in the brain, brainstem, and spinal cord, resulting in fatal paralysis	105400 608030	Janssen et al. (2010); Lazo-Gomez et al. (2013); Wang et al. (2008)
Kleefstra syndrome	EHMT1 (GLP, KMT1D)	Reduced H3K9 MTase activity due to mutations in *EHMT1*	Severe mental retardation, hypotonia, brachy(micro)cephaly, epileptic seizures, flat face with hypertelorism, synophrys, anteverted nares, everted lower lip, carp mouth with macroglossia, and heart defects	610253	Kleefstra et al. (2006)
Weaver syndrome	EZH2 (KMT6)	Reduced H3K27me3 MTase activity due to mutations in *EZH2*	Pre- and postnatal overgrowth, accelerated osseous maturation, characteristic craniofacial appearance, and developmental delay	277590	Gibson et al. (2012); Tatton-Brown et al. (2011); Tatton-Brown et al. (2013)
Sotos syndrome	NSD1 (KMT3B)	Reduced H4K20 and H3K36 methylation due to mutations in *NSD1*	Excessively rapid growth, acromegalic features, and a nonprogressive cerebral disorder with mental retardation	117550	Kurotaki et al. (2002)
Kabuki syndrome	MLL2 (KMT2B); UTX (KDM6A)	De-regulated chromatin state due to mutations in one or both *MLL2* and *KDM6A*	Mental retardation and postnatal dwarfism, a peculiar facies characterised by long palpebral fissures with eversion of the lateral third of the lower eyelids	147920 300867	Lederer et al. (2012); Miyake et al. (2013); Ng et al. (2010)
X-linked mental retardation	PHF8; JARID1C (SMCX, KDM5C)	Reduced histone de-methylase activity due to mutations in *PHF8* and *JARID1C*	Mental retardation, cleft lip and palate	300263 300534	Iwase et al. (2007); Jensen et al. (2005); Laumonnier et al. (2005)
Brachydactyly mental retardation syndrome	HDAC4	Reduced HDAC activity due to haploinsufficiency of *HDAC4*	Short stature, stocky build, mental retardation, brachymetaphalangia, and eczema	600430	Williams et al. (2010)
Chromatin remodeling					
CHARGE syndrome	CHD7	De-regulated chromatin state via CHD7 haploinsufficiency	Coloboma, Heart malformations, Atresia of the choanae, Retardation of growth, Genital hypoplasia, and Ear abnormalities	214800	Vissers et al. (2004); Zentner et al. (2010)
ATR-X syndrome; Juberg–Marsidi syndrome; Sutherland–Haan syndrome; Smith–Fineman–Myers syndrome	ATRX (RAD54L)	De-regulated chromatin state via mutation in a SNF2-type helicase	α-Thalassemia, mental retardation, facial and skeletal abnormalities, urogenital abnormalities, microcephaly	301040 309590 309470 309580	Gibbons et al. (1995); Gibbons and Higgs (2000); Gibbons et al. (2008)
Coffin-Siris syndrome; MRD12; MRD14; MRD15; MRD16	SMARCB1 (BAF47, SNF5L1); SMARCA4 (BRG1, BAF190A, SNF2B); SMARCA2 (BRM, BAF190B, SNF2A); ARID1A (BAF250A); ARID1B (BAF250B)	De-regulated chromatin state via mutations in SWI/SNF remodelers	Developmental delay, intellectual disability, speech impairment, absent or hypoplastic fifth fingernails or toenails, hypotonia, hirsutism	135900 614562 614607 614608 614609	Tsurusaki et al. (2012)
Cerebro-oculo-facio-skeletal syndrome (COFS); Cockayne syndrome	ERCC6 (CSB)	De-regulated chromatin state via *ERCC6* mutations	Postnatal growth failure, neurological degeneration, cataracts, progressive joint contractures, dysmorphic features and premature death, UV sensitivity	214150 133540	Mallery et al. (1998)

A list of selected diseases associated with defects in chromatin regulation. The affected gene(s) and epigenetic mechanisms involved are described, as are the symptoms. Common synonyms for gene names are given in parentheses

^a^Reference numbers for individual diseases can be found in the Online Mendelian Inheritance in Man catalogue: http://www.ncbi.nlm.nih.gov/omim

### DNA modifications

The major form of DNA modification is methylation of the DNA at the 5-position of cytosine in CpG dinucleotides. The methyl mark is placed by enzymes known as DNA methyltransferases (DNMTs) that transfer a methyl group from S-adenosyl methionine to DNA (for a summary of the functions of DNA methylation and DNMTs see reviews by Li and Zhang 2014 and Jurkowska et al. 2011). In mammals, DNA methyltransferases can be grouped into de novo DNMTs (DNMT3A and DNMT3B) and maintenance DNMTs (DNMT1). De novo DNMTs establish DNA methylation patterns during embryonic development and are highly expressed in embryonic stem (ES) cells and downregulated in differentiated tissues. The catalytically inactive DNMT3L acts as a general co-factor for DNMT3A and DNMT3B. DNMT1, on the other hand, has a preference for hemi-methylated DNA. It is thought to follow the replication fork via interactions with PCNA (Chuang et al. 1997) and UHRF1 (Bostick et al. 2007; Sharif et al. 2007), in restoring DNA CpG methylation on the hemi-methylated DNA after synthesis of the daughter strand.

DNA methylation is generally associated with repressed regions of the genome. CpG dinucleotides constitute only around 1 % of the mammalian genome with the majority being methylated. CpG islands—regions of highly elevated CpG content—are associated with about 60 % of human gene promoters and are usually unmethylated in normal cells, although around 4 % become methylated in a tissue-specific manner during early development which generally leads to silencing of the associated genes (Borgel et al. 2010; Shen et al. 2007). However, gene body DNA methylation is coupled to transcriptional activation in ubiquitously expressed genes and might correlate with elongation efficiency (Laurent et al. 2010; Lister et al. 2009). Many repetitive elements present in the genome are silenced through DNA methylation to prevent aberrant expression that could cause chromosomal instability, translocations and gene disruption due to transposition events. DNA methylation is also involved in imprinting, which is the silencing of autosomal genes in a parent specific pattern. Imprinted genes tend to have a closely associated imprinting control region (ICR) the methylation status of which dictates whether the paternal or maternal allele is expressed (for a review of the mechanisms of imprinting see Sanli and Feil 2015). A similar gene-dosage reduction is seen in X chromosome inactivation in females (for details see review by Chaligne and Heard 2014). DNA methylation is further linked to nuclear organisation (reviewed in Pombo and Dillon 2015), concentrating in dense silenced chromatin regions known as heterochromatin.

CpG methylation exerts its repressive effects by blocking DNA-binding proteins such as transcription factors from accessing DNA or by recruiting proteins that contain methyl-CpG binding domains (MBD). One example is MeCP2 which, in addition to its MBD, contains a transcriptional-repression domain (TRD) that recruits other co-repressors such as Sin3a and histone deacetylases as well as chromatin remodelers that maintain the silenced chromatin state (Jones et al. 1998; Nan et al. 1998).

DNA methylation has traditionally been regarded as a stable modification with few mechanisms for its removal. However, over recent years, oxidised forms of 5-methyl cytosine (5mC)—hydroxy-methyl cytosine, formyl-methyl cytosine, and carboxy-methyl cytosine—were identified (reviewed in Li and Zhang 2014). The identification of these marks and the enzymes involved in their formation (such as the TET proteins) suggests that these oxidation products may form intermediates in the removal of the repressive 5mC mark. Furthermore, the oxidised forms of 5mC may also have independent roles in directly regulating gene expression (discussed in Wang et al. 2014).

### Histone modifications

Histone proteins are predominantly globular proteins with flexible N- and C-terminal tails that contain a high percentage of basic lysine and arginine residues protruding from the nucleosome particle. The amino acid residues on these flexible tails are extensively post-translationally modified. Numerous modifications exist such as lysine or arginine methylation, lysine acetylation and ubiquitylation, serine or threonine phosphorylation and ADP-ribosylation to name but a few. These PTMs rarely occur in isolation and it is the position, extent and context of the modifications that mediate their regulatory effects on chromatin (for a detailed review of histone modifications and their functions, see Bannister and Kouzarides 2011).

Histone PTMs are deposited by modifying enzymes (collectively known as epigenetic “writers”) such as methyltransferases (MTase) and acetyltransferases whilst de-modifying enzymes (or “erasers”) remove these marks. These opposing activities enable a highly dynamic regulation of gene expression as modifications can be added or removed depending on whether a particular writer or eraser is recruited to a specific location of the genome. Histone PTMs act as docking sites for epigenetic “reader” proteins that contain modification-specific binding domains. These binding domains, such as bromodomains that can recognise acetylated lysines, or PHD domains that can bind methylated lysines, are integral in directing epigenetic effector proteins to chromatin (discussed in Yun et al. 2011). Histone PTMs are thought to form a code that enables highly specific recruitment of epigenetic factors that mediate distinct downstream events to particular regions of chromatin (Jenuwein and Allis 2001; Strahl and Allis 2000). Histone modifications can also directly affect chromatin structure. Histone acetylation and phosphorylation effectively reduce the positive charge of histones and potentially affect the electrostatic interactions between histones and DNA or between neighbouring nucleosomes which can lead to a de-condensation of chromatin. This is supported by evidence that histone acetylation is associated with active open chromatin that is sensitive to DNase digestion (Hebbes et al. 1994) and that acetylation of histone H4 at lysine 16 (H4K16) diminishes the compaction of chromatin arrays in vitro (Shogren-Knaak et al. 2006).

Many histone PTMs correlate with gene expression (Fig. 1). For instance, lysine acetylation is generally associated with actively transcribed chromatin (termed euchromatin) along with methylation of histone H3 lysine 4, which marks promoter and enhancer regions, and H3K36 methylation that is found in active gene bodies. H3K9 and H3K27 methylation, on the other hand, are marks predominantly associated with silenced heterochromatic regions of the genome (Roadmap Epigenomics et al. 2015).

### Chromatin remodeling

A third mechanism involved in chromatin regulation is the control of nucleosome positioning by chromatin remodeling factors. Together with histone chaperones, chromatin remodelers shape chromatin architecture by relocating nucleosomes along the DNA, creating nucleosome free regions through eviction of histones, or by facilitating the deposition or exchange of new histones and histone variants. These factors allow access of the transcription machinery to the normally condensed genomic DNA and thereby control gene expression and processes such as DNA replication and repair. A common feature of all chromatin remodelers is the presence of an enzymatic ATPase subunit, which allows them to utilize the energy released from ATP hydrolysis to reposition nucleosomes. Chromatin remodelers are classified into four families (the SWI/SNF, ISWI, CHD and INO80/SWR1 families) based on their subunit composition and their modes of nucleosome remodeling. For further details regarding histone chaperones and chromatin remodelers, we refer the reader to two recent reviews (Gurard-Levin et al. 2014; Narlikar et al. 2013).

## Deregulation of chromatin in disease

**Fig. 3:**
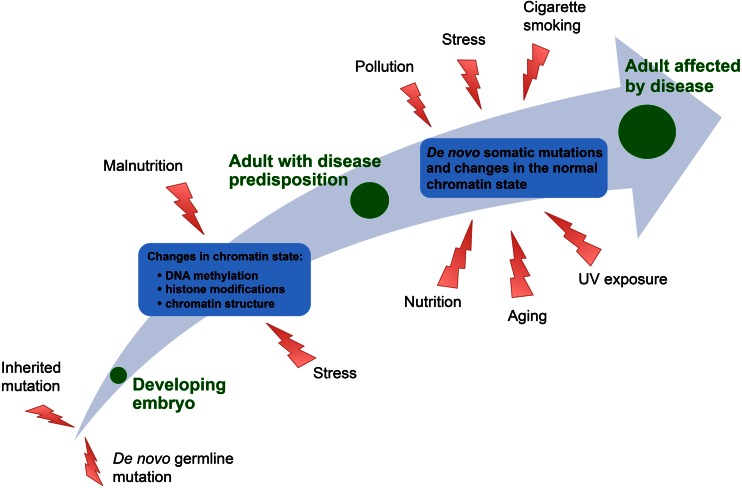
Factors involved in the formation of epigenetic diseases. Diseases caused by chromatin deregulation can arise through environmental stress, either during foetal development or later in life, or by mutations in genes encoding chromatin regulators. These mutations can be heritable or acquired de novo and can predispose an adult to disease in response to environmental stresses

Given the importance of chromatin modifications and nucleosome remodeling in gene regulation, it is apparent that loss or alteration of their functions can have detrimental consequences. Epigenetic deregulation has been the subject of an increasing number of studies into a wide range of pathologies from cancer to neurological disorders. Here, we will focus on the different mechanisms of chromatin regulation and discuss how defects in the epigenetic machinery can lead to disease (Figs. 2 and 3).

### Deregulated DNA methylation

DNA methylation is generally associated with transcriptionally repressed chromatin. Various mechanisms can lead to DNA hypo- or hyper-methylation causing aberrant gene expression patterns underlying many diseases.

#### Deregulated gene expression

Several neurodegenerative disorders have been found to involve aberrant DNA methylation patterns at CpG-rich regions of disease-associated genes (discussed in Lu et al. 2013).

In Parkinson’s disease (PD), progressive loss of substantia nigra dopaminergic neurons and striatal projections causes muscular rigidity and tremors. Fibrillar aggregates of misfolded α-synuclein form Lewy bodies that accumulate at sites of neuronal loss. There is evidence for altered DNA methylation in the promoter of the *SNCA* gene (encoding α-synuclein) with decreased methylation in intron 1 that might lead to increased expression of α-synuclein and resulting formation of aggregates (Jowaed et al. 2010). Methylation is generally reduced in the substantia nigra of PD patients resulting in an altered chromatin landscape (Matsumoto et al. 2010). Further evidence indicates that DNMT1 expression is reduced in PD patient brains and in α-synuclein transgenic mice, with DNMT1 thought to be sequestered in the cytoplasm by α-synuclein, leading to hypo-methylated CpGs (Desplats et al. 2011). Furthermore, hypo-methylation of the TNF-α gene promoter in the substantia nigra results in TNF-α overexpression and apoptosis of neuronal cells (Pieper et al. 2008).

Aberrant DNA methylation patterns and mis-expression of disease-associated genes have also been found in other neurodegenerative disorders such as Alzheimer’s and Huntington’s disease and amyotrophic lateral sclerosis (see Lu et al. 2013). An interesting aspect is that DNA methylation status could impact on the stability of CTG/CAG trinucleotide repeats and contribute to the expansion of the poly-glutamine stretch within the huntingtin protein that causes Huntington’s disease (Behn-Krappa and Doerfler 1994; Gorbunova et al. 2004).

#### Deregulated imprinting

Aberrant methylation at ICRs of imprinted regions of the genome can lead to deregulated expression of imprinted genes. Two such diseases, Prader-Willi syndrome and Angelman syndrome, originate from a loss of regulation at the ICR at q11–q13 on chromosome 15 (see Cassidy and Schwartz 1998 and Clayton-Smith and Pembrey 1992).

In Prader-Willi syndrome, loss of expression of several paternal genes in this region leads to infantile hypotonia, hyperphagia resulting in obesity, and mental retardation. Angelman syndrome on the other hand arises from the loss of maternally expressed genes in this region, specifically *UBE3A* (Kishino et al. 1997; Matsuura et al. 1997), leading to more severe mental retardation. Both are thought to involve cis-acting mutations preventing the re-setting of the imprinting signal specifically for this locus as other ICRs tend to be unaffected. In some cases of Angelman syndrome, MeCP2 mutations have been found, indicating a role for this methyl-CpG binding protein in imprinting at the 15q11 locus (Hogart et al. 2007; Samaco et al. 2005).

#### Repeat expansion/deletion

Genomic repeat sequences are generally methylated once above a threshold length to ensure transcriptional silencing. Repeats therefore tend to be located within heterochromatic regions. Some diseases arise from expansion or loss of repeats, leading to aberrant locus-specific CpG methylation that can affect gene expression of nearby genes.

Fragile X syndrome is caused by the silencing of the *FMR1* (Fragile X Mental Retardation 1) gene. An expansion of CGG tri-nucleotide repeats in the 5′UTR of *FMR1* has been associated with disease onset (Jin and Warren 2000). Expansion to >200 copies of this triplet repeat induces hyper-methylation in the *FMR1* promoter region and leads to transcriptional silencing (Sutcliffe et al. 1992; Wang and Griffith 1996). Examination of the genome-wide DNA methylation state indicates no other abnormally DNA methylated loci (Alisch et al. 2013).

In a related mechanism, Facioscapulohumeral muscular dystrophy (FSHD) originates from a loss of repeats rather than repeat expansion. FSHD is an autosomal dominant disease that results in a progressive wasting of facial, upper arm and shoulder girdle muscles following the de-repression of genes at the *FSHD* locus in the sub-telomeric 4q35 region of chromosome 4. De-repression is caused by a reduced copy number of neighbouring D4Z4 repeats. A reduced number of repeats to <11 leads to loss of polycomb-mediated repression and expression of a ncRNA called DBE-T that is located proximal to the D4Z4 repeats. DBE-T is produced only in FSHD patients and seems to act in a positive feedback loop to coordinate 4q35 de-repression by recruiting the tritorax group protein Ash1L. This ultimately results in the expression of neighbouring genes in the FSHD locus and onset of the disease (Cabianca et al. 2012).

#### Mutations in DNMTs

The importance of DNA methylation suggests that defects in the machinery that deposit this modification will have deleterious consequences, and indeed DNMTs are found to be mutated in several diseases.

One example is ICF (immunodeficiency, centromere instability and facial anomalies) syndrome, a recessive autoimmune disease caused by a mutation in both alleles of *DNMT3B* (Hansen et al. 1999; Xu et al. 1999). Approximately 60 % of ICF patients have mutations in *DNMT3B* which is used in diagnosis of the disease. These mutations are usually missense mutations either in the catalytic domain (see Ehrlich et al. 2008) or in the regions that mediate binding to DNMT3L, the non-active co-factor required for DNMT3B activity (Jiang et al. 2005; Xie et al. 2006). This results in a loss of catalytic activity but is not lethal, indicating that these mutations might alter the specificity of the enzyme so that it no longer recognises and methylates specific sequences whilst enabling other DNMT targets to remain unaffected. Indeed, HPLC analysis of the methylation level indicates only a 7 % hypo-methylation in the brain of ICF patients (Ehrlich 2003).

ICF patients show DNA rearrangements targeted to juxtacentromeric heterochromatin regions of chromosomes 1, 9 and 16. These pericentromeric regions display marked DNA hypo-methylation in repeat sequences (satellite 2 and 3, alpha-satellite, Alu, D4Z4 and NBL2 repeats), as observed in lymphocytes, that is thought to result in the instability of this constitutive heterochromatin (Ehrlich et al. 2008). Despite generally unchanged global DNA methylation levels there are some changes to expression of genes involved in immune response function and neurodevelopment. The direct causal effect of this is not fully understood but changes observed in ICF patients include CpG hypo-methylation at DNMT3B target genes, and loss of the repressive tri-methyl H3K27 (H3K27me3) and gain of the activating H3K4me3 mark (Jin et al. 2008).

#### Mutations in methyl-CpG binding proteins

The ability to recognise methylated regions of the genome is important for establishing transcriptional repression in response to DNA methylation.

The major factor involved is methyl-CpG-binding protein MeCP2 that binds methylated DNA through its MBD domain (see Bird 2008). Mutations in *MECP2* cause Rett syndrome, a form of postnatal mental retardation involving the regression from relatively normal cognitive function to a loss of speech and motor skills within the first few years of childhood (Amir et al. 1999). It is an X-linked neurological disease affecting almost exclusively females where patients tend to be heterozygous for the mutated allele (random X inactivation results in a mosaic phenotype in females). The mutated allele is almost exclusively acquired de novo in the germ cells with mutations varying from single missense mutations to truncated or mis-folded versions of the protein. Missense mutations frequently occur within the MBD region, thus preventing the protein from binding methylated DNA. Other mutations also target the TRD domain that is involved in binding and recruiting co-repressors such as Sin3a and HDAC1 (Lyst et al. 2013). The resulting loss of repression of methylated genomic regions directly contributes to disease onset.

Knockout mouse models for *MECP2* have confirmed the cause and phenotype for Rett syndrome (Chen et al. 2001; Collins et al. 2004). However, there is striking evidence that these defects could be reversible. The lack of neuronal cell death suggests that Rett syndrome is a neurodevelopmental disease rather than being neurodegenerative and it was hypothesised that adding back functional MeCP2 may rescue the phenotype. Restoration of the gene was indeed found to rectify *MECP2* KO mice indicating a principle of reversibility for neurodevelopmental disorders (Bird 2008; Cobb et al. 2010; Guy et al. 2007)

### Deregulated histone modifications

A major mechanism in the epigenetic regulation of chromatin is the post-translational modification of histone proteins. Mutation or deregulated expression of histone modifying or de-modifying enzymes can lead to altered deposition of modification marks. As discussed above, loss in the regulatory control of histone modifications has the potential to affect gene expression and can directly impact on chromatin structure. Histone modifiers can induce global changes in gene expression patterns in response to upstream signals. Therefore, perturbations in their ability to process these signals can have knock-on effects in gene regulatory networks.

#### Aberrant histone acetylation

A histone mark associated with active gene expression is lysine acetylation. Acetyl groups are transferred to lysines by histone acetyltransferases (HATs) and removed by histone de-acetylases (HDACs) (for details see Bannister and Kouzarides 2011). Neurodegenerative and other neurological disorders are generally characterised by histone hypo-acetylation due to reduced HAT activity or increased HDAC activity.

Rubinstein–Taybi syndrome is an example of an autosomal dominant disorder resulting in cognitive dysfunction that is associated with loss of HAT activity (Petrij et al. 1995). It is a genetically heterogeneous disease, with 55 % of patients displaying mutations in the *CBP* (CREB binding protein) and 3 % in the *EP300* gene whereas the remainder are uncharacterised (Roelfsema et al. 2005). CBP and p300 are both HATs with co-activator function in gene expression. Heterozygous *CBP*^*+/−*^ mice display a reduction in H2B acetylation by more than 30 % and failure in long-term memory function mirroring the symptoms of Rubinstein–Taybi syndrome (Alarcon et al. 2004).

Another pathology involving deregulated histone acetylation is amyotrophic lateral sclerosis (ALS). This is a fatal neurodegenerative disease resulting from the death of upper and lower motor neurons. Familial ALS stems from mutations in *SOD1* (superoxide dismutase) but this only accounts for approximately 10 % of patients whereas sporadic ALS is vastly uncharacterised (see Lazo-Gomez et al. 2013). A major cause of the disease appears to be the formation of cytoplasmic aggregates of mis-folded FUS protein. FUS is an RNA-binding protein with roles in splicing, nuclear transport and translation but it might also have a role in transcription (Ishigaki et al. 2012; Schwartz et al. 2012). FUS binds CBP, strongly inhibiting HAT activity and negatively regulates CREB target genes (Wang et al. 2008). Furthermore, there is an alteration in HDAC levels in the brain and spinal cord of ALS patients with decreased HDAC1 and increased HDAC2 mRNA levels (Janssen et al. 2010). This indicates that aberrant histone acetylation seems to play a role in ALS.

#### Aberrant histone methylation

Lysine and arginine methylation of histones is another important mechanism in chromatin regulation and several diseases have been associated with aberrant histone methylation patterns.

Kleefstra syndrome is associated with haplo-insufficiency of the histone MTase EHMT1 that deposits H3K9me2 marks (Kleefstra et al. 2006; Ogawa et al. 2002). Its symptoms include intellectual disability and muscular hypotonia. Mutations involve a range of deletions, from large to very small, in the *EHMT1* locus leading to a loss of its MTase activity. Indeed, in mouse models the loss of *EHMT1* results in reduced learning and memory, hypotonia and cranial abnormalities (Balemans et al. 2014; Schaefer et al. 2009). EHMT1 was found to interact with several transcriptional repressors, including E2F6 and REST. Loss of targeted H3K9-methylation may therefore be important for disease onset (Ogawa et al. 2002; Roopra et al. 2004; Tahiliani et al. 2007).

Other diseases caused by loss of histone MTase activity are Weaver and Sotos syndrome (Tatton-Brown et al. 2013). Both are mostly sporadic diseases and have similar traits, including intellectual disability and tall stature but interestingly see mutations in alternative MTase enzymes. Weaver syndrome displays mutations in the *EZH2* gene, the MTase subunit of the PRC2 complex, that primarily cluster to the catalytic SET domain and result in reduced H3K27me3 levels (Gibson et al. 2012; Tatton-Brown et al. 2011). In Sotos syndrome, however, mutations primarily affect *NSD1* (Kurotaki et al. 2002) which is thought to abrogate methylation of H3K36 as well as H4K20 (Rayasam et al. 2003). The similar phenotypes indicate a common pathway between these two seemingly separate chromatin modifiers.

A similar situation is found in Kabuki syndrome, which can result from loss-of function mutations in either the H3K4 MTase MLL2 or the H3K27 de-methylase KDM6A (Lederer et al. 2012; Miyake et al. 2013; Ng et al. 2010). In both cases, mutations presumably lead to reduced active marks and increased repressive marks in the diseased state, possibly causing reduced gene expression at common target loci since MLL2 and KDM6a have been show to reside in the same protein complex.

#### Aberrant histone de-modifiers

Apart from KDM6A there are examples of several other histone de-modifying enzymes being associated with mental retardation syndromes (discussed in Kramer and van Bokhoven 2009).

PHF8 is a H3K9 de-methylase that binds to tri-methylated H3K4 and localises to transcription start sites. Mutations in its de-methylase domain have been associated with X-linked mental retardation (XLMR) (Laumonnier et al. 2005). PHF8 interacts with zinc-finger protein ZNF711 (Kleine-Kohlbrecher et al. 2010), another XLMR protein, and both proteins share common target genes including *KDM5C* (also known as SMCX or JARID1C). KDM5C is a H3K4 de-methylase that binds methylated H3K9 through a PHD domain and missense mutations in *KDM5C* itself have been associated with XLMR (Iwase et al. 2007; Jensen et al. 2005). XLMR exemplifies that, although individual epigenetic effectors have specific functions, the overriding effect on gene expression programmes is dictated by the interplay between several readers, writers and erasers and upsetting this balance can lead to disease.

Deregulation of HDACs is known to contribute to cancer formation, but HDAC mutations are also linked to other diseases. *HDAC4* haplo-insufficiency has been linked to brachydactyly mental retardation syndrome, causing developmental delays, behavioural problems and sleep disturbance, and craniofacial and skeletal abnormalities (Williams et al. 2010). This disease is caused by deletions at the 2q37.3 locus (a region encoding *HDAC4*) or de novo mutations of *HDAC4* in the germ line that result in reduced HDAC activity.

### Deregulation of chromatin remodeling

In recent years, several complex disease syndromes have been linked to mutations in genes encoding chromatin remodeling enzymes. Prominent examples are ATRX syndrome, α-thalassemia, Sutherland–Haan syndrome, Juberg–Marsidi and Smith–Fineman–Myers syndrome all of which are caused by mutations in the *ATRX* gene. All syndromes share common phenotypes including mental retardation, mild α-thalassemia and facial, skeletal and urogenital abnormalities during development (discussed in Gibbons and Higgs 2000). The ATRX mutations concentrate in two conserved domains, the ADD and the helicase domains (Gibbons et al. 2008). Although the precise disease mechanisms are still unknown, all disorders display changes in methylation patterns at DNA repeats (Gibbons et al. 2000). *ATRX* encodes an ATP-dependent type II helicase of the SNF2 family that is highly expressed during development. ATRX primarily localises to pericentromeric heterochromatin, rDNA repeats, telomeres and PML bodies (Gibbons et al. 2000; McDowell et al. 1999; Xue et al. 2003). It associates with two key heterochromatin proteins, HP1 (Lechner et al. 2005) and EZH2 (Cardoso et al. 1998), and co-operates with the histone chaperone DAXX to deposit H3.3 into chromatin (Lewis et al. 2010; Xue et al. 2003). ATRX appears to recruit methyltransferases to repetitive heterochromatic sequences possibly through binding to transcribed repeat RNAs (Sarma et al. 2014). Further studies showed an association with G-rich tandem repeats linking it to the potential recognition of G-quadruplex structures in vivo, which are prevalently found in telomeres (Law et al. 2010). Indeed, loss of ATRX causes telomere instability. The notion that ATRX plays a role at telomeres is also supported by the finding that it concentrates at late replicating chromatin and that loss of ATRX causes delays in completing DNA replication (Huh et al. 2012). The exact molecular mechanisms and the link between the various observed abnormalities caused by ATRX mutations remain to be established.

A second example of a disease involving defective chromatin remodeling is CHARGE syndrome, which is caused by heterozygous mutations in the gene encoding CHD7 (Vissers et al. 2004). CHARGE syndrome is characterised by developmental retardation with specific heart defects, genital hypoplasia, vestibular disorders, ear abnormalities and deafness. The molecular causes underlying the disorder are not yet understood and the disease is associated with many phenotypes with the clinical variability between patients and even between family members carrying the same mutations being very broad (Zentner et al. 2010). Several CHD7 mutations identified in CHARGE syndrome were found to affect its nucleosome remodeling activity (Bouazoune and Kingston 2012). CHD7 recognises all methylated forms of histone H3K4 and localises to regions of active transcription and enhancer elements (Schnetz et al. 2009). The binding pattern and the functionality of the protein suggest that CHD7 promotes transcription by binding enhancer elements and transiently looping chromatin to transcription start sites.

## Extrinsic and intrinsic factors influencing the epigenome

The main cause for the diseases discussed above are genetic mutations that lead to detrimental changes in the chromatin landscape. The plasticity of the epigenome also means that it can respond to stimuli from the environment that affect transcriptional regulation without changes in the DNA sequence. Exposure to environmental stresses and cross-talk/feedback between the epigenetic machinery and the environment can reinforce faulty gene expression patters leading to disease (Fig. 3).

The nature versus nurture conundrum has sparked studies attempting to understand how the environment influences the susceptibility, progression and outcome of diseases. Research in the human population focuses on monozygotic twins, a fortunate model for genetically identical individuals that show significantly different gene-expression phenotypes and often different susceptibilities to diseases such as mental disorders, diabetes, obesity, allergies and longevity. It has been demonstrated that twins vary in their epigenomes (Fraga et al. 2005). The Epitwin project (http://www.epitwin.eu) plans to trace differences in the methylation patterns of 20 million CpG islands in 5000 monozygotic twins with the goal of explaining why twins do not develop the same diseases and how the epigenome responds to external stimuli.

### Intrinsic factors

Genetic mutations in chromatin regulators are the major causes of epigenetic diseases. They can either be inherited (as seen in hereditary diseases) or occur de novo in the germline of the parents, during embryogenesis or somatically (Fig. 3). Apart from genetic factors, there also appear to exist naturally occurring processes that perturb chromatin modification patterns without altering the DNA sequence.

After each cell division, the DNA methylation pattern is passed on to the daughter cells, a process safeguarded by DNMTs. Strikingly, global methylation levels tend to decrease in many tissues with increasing age. This large-scale hypomethylation appears to be stochastic and mainly localised to repetitive sequences such as SINEs and LTRs possibly leading to genomic instability (Berdasco and Esteller 2012; Ronn et al. 2008; Wilson and Jones 1983; Zampieri et al. 2015). In addition, there are reports of ageing-related hyper-methylation events. These are relatively sparse and mostly targeted to specific CpG island promoters resulting in silencing of associated genes (discussed in Zampieri et al. 2015). The hypo- and hyper-methylation events observed during ageing resemble methylation patterns seen in cancer and could promote oncogenesis by silencing tumour suppressors or predispose aged individuals to other age-related diseases. For example, in Alzheimer’s disease amyloid-β protein deposition in the brain has been connected with cytosine de-methylation at the *APP* gene leading to its increased expression (Tohgi et al. 1999), and the COX7A1 gene, which plays a role in glucose metabolism was found to become highly methylated with age (Ronn et al. 2008). This might increase the risk of insulin resistance and contribute to the development of type-2 diabetes commonly affecting elderly individuals.

Interestingly, levels of histone modifications were also found to be deregulated with age, and in the premature ageing disease Hutchinson–Gilford Progeria Syndrome the deregulation of histone modifications recapitulates some of the epigenetic alterations observed in normal ageing. Specifically, there is a reduction of H3K9me3 and H3K27me3 paralleled with a downregulation of EZH2, and a spread of H4K20me3 (see Prokocimer et al. 2013).

### Influence of the environment

#### Cigarette smoking

Exposure to cigarette smoke has been correlated with an increased risk of epigenetic diseases. Cigarettes contain more than 500 chemicals, and when smoked 4,000 chemicals are produced. Many of these have been linked to development of emphysema, heart diseases and cancer. Promoter hyper-methylation of tumour-suppressor genes was demonstrated in non-tumourigenic lung tissues of smokers, but not in the corresponding tissue of non-smokers (Zochbauer-Muller et al. 2002). Shenker et al. (2013) also identified hypo-methylated loci associated with smokers compared to former and non-smokers (Shenker et al. 2013). The effect of smoking on DNA methylation varies between ethnic groups due to different genetic and environmental backgrounds causing different susceptibility to diseases. For example, the DNA methylation level of the lung cancer-associated *AHRR* locus was lower for European smokers than for South Asian smokers (Elliott et al. 2014).

#### Nutrition

The link between nutrition and epigenetics is exceptionally important as nutrients and bioactive food components directly influence epigenetic events and impact on gene expression at the transcription level (discussed in Jimenez-Chillaron et al. 2012). Nutrients such as vitamin B-12, choline, folate and methionine affect histone and DNA methylation through their involvement in 1-carbon metabolism and were shown to be capable of increasing DNA methylation levels at specific loci in animal models (Waterland 2006) and humans (Waterland et al. 2010). Histone acetylation is linked to energy metabolism and responds to intracellular glucose levels through the availability of the HAT co-factor acetyl coenzyme A (Takahashi et al. 2006; Wellen et al. 2009). Oncogenic Kras and Akt signaling were shown to cause increased histone acetylation in tumours through stimulation of ATP-citrate lyase, the key enzyme for cytoplasmic acetyl coenzyme A synthesis (Lee et al. 2014). Substances occurring in some foods, like butyrate in cheeses, diallyl disulphide in garlic and sulphoraphane in broccoli, are natural HDAC inhibitors. Therefore, these substances have been proposed in cancer chemoprevention as possible mediators for induction of apoptosis by increasing acetylation and de-repressing genes such as *P21* and *BAX* (discussed in Dashwood and Ho 2007).

In development, critical epigenetic events occur at specific time windows during which the entire epigenome is reprogrammed and post-translational marks are erased and then re-established. Nutrition was shown to influence the epigenome during embryonic development and early childhood with potential pathologies later in life (see review by Jimenez-Chillaron et al. 2012). Maternal nutritional status and particular diets can affect chromatin structure in embryonic tissues depending on the developmental stage. In the case of foetal starvation, there is high correlation with type-2 diabetes susceptibility in adulthood (Park et al. 2008; Sandovici et al. 2011). If the epigenome of developing germ cells is affected, these conditions can also be passed on between generations. Recent mouse studies demonstrate that the in utero nutritional environment of embryos during development alters the germline DNA methylome of adult males in a locus-specific manner. Although the differential methylation pattern is not maintained in the tissues of the following generations, perturbed locus-specific gene expression persists (Radford et al. 2014).

#### Developmental stress

Stress exposure in early life (ELS) has been associated with higher risk of mental pathologies such as bipolar disorder, schizophrenia, depression, anxiety and posttraumatic stress disorder (discussed in Gershon et al. 2013). The effects of poor maternal care on offspring at an epigenetic level have been studied in mouse models, showing that gene expression and methylation levels are affected in pups and that the phenotype can be rescued when the same pups are fostered by caring mothers (Francis et al. 1999). Genes that regulate the hypothalamic pituitary adrenal axis, which is functionally responsible for the homeostatic response during stress, were found to be deregulated at an epigenetic level in animals and humans affected by ELS. Specifically, the expression levels of neurotrophic factor BDNF were reported to be lower in ELS mouse models (Roth et al. 2009) and in patients affected by mental disorders and exposed to childhood trauma (Ernst et al. 2005; Hashimoto et al. 2005). This coincides with increased methylation levels at the BDNF locus and its receptor TrkB (Perroud et al. 2013). In addition, hyper-methylation of the NR3C1 gene was found in ELS animal models and in adults with childhood trauma. This was reversed by changing the fostering of the pups and by the use of HDAC inhibitors (Weaver et al. 2006) underscoring the value of drugs targeting reversible epigenetic events (see below).

#### Environmental factors

Chemicals and pollutants found in the environment have been associated with epigenetic pathologies. Heavy metal ions such as cadmium, chromium and nickel (Salnikow and Costa 2000; Shiao et al. 2005) were found to reduce methylation levels at genetic loci by inhibiting the activity of DNMTs (Poirier and Vlasova 2002). In addition, compounds that until recently were designated for different usage such as diethylstilbestrol (Veurink et al. 2005), a drug previously used to prevent miscarriages, bisphenol A (Maffini et al. 2006), used in the plastics industry, and vinclozolin (Anway et al. 2006), a fungicide used in vineyards, were found to be endocrine disruptors and have been convincingly related to altered DNA methylation at specific promoters, developmental disorders and tumourigenesis.

## Epigenetic deregulation in complex diseases

Given the enormous importance of epigenetic modifications for gene regulation, development and tissue homeostasis, it is not surprising that disruption of epigenetic mechanisms causes pathologies affecting various tissues and organs. Certain diseases originate from mutation or deregulation of several genes, often in combination with environmental factors. Recent technological developments have enabled the large scale sequencing of patient genomes and have linked mutations in epigenetic regulators to multifactorial diseases such as congenital heart diseases, mental disorders and cancer. Patients with polygenic diseases often display alterations in common pathways mostly caused by de novo mutations in functionally related genes.

Sequencing of patients with congenital heart defects has revealed an extensive list of mutations in H3K4 methylation regulators including MLL2, KDM6A, KDM5A, KDM5B, CHD7 and WDR5 (Zaidi et al. 2013). Mutations in MLL2 and KDM6A are also implicated in Kabuki syndrome (Lederer et al. 2012; Miyake et al. 2013; Ng et al. 2010), whilst mutations in CHD7 are implicated in CHARGE syndrome (Vissers et al. 2004). Both diseases have heart defects as common symptoms.

De novo mutations in CHD8 and other chromatin remodelers including CHD7 have been associated with Autism Spectrum Disorders (ASD), neurodevelopmental disorders that affect social interaction, communication and behaviour (summarised in Krumm et al. 2014). Interestingly, CHD8 interacts with CHD7 (Batsukh et al. 2010) and the majority of CHARGE syndrome patients suffer from ASD (Johansson et al. 2006), demonstrating that mutations in related epigenetic pathways can result in different syndromes with shared symptoms depending on the context and nature of a particular mutation.

Cancer has traditionally been regarded as being mainly caused by genetic defects. However, over the last two decades, the role of epigenetic deregulation as a hallmark of cancer has been widely acknowledged. Genome-wide hypo-methylation is commonly observed in cancer (Feinberg and Vogelstein 1983) and has been linked to chromosomal instability (Eden et al. 2003). Epigentic silencing by de novo promoter methylation has been recognised as an additional pathway in Knudson’s two hit hypothesis postulating that two events are necessary for the inactivation of a tumour suppressor. Clear examples of this are the de novo methylation of gene promoters for the *Rb, CDKN2A* (p16^INKa^) and *CDKN2B* (p15^INKb^) tumour suppressors (see Jones and Laird 1999). Mutations in coding sequences of epigenetic regulators are also often found in tumours, e.g. the DNA-modifying enzymes DNMT3A and TET2 are frequently mutated in leukaemia, lymphomas and myeloid cancers (Delhommeau et al. 2009; Ley et al. 2010). Furthermore, histone modifying enzymes such as HATs and histone MTases or de-modifying enzymes such as HDACs and histone de-methylases are deregulated in cancer. Observed lesions include missense mutations, truncations, deletions and amplifications leading to altered expression levels, and—especially for the H3K4 methyltransferase MLL and the HATs p300 and CBP—the expression of fusion proteins resulting from chromosomal translocations (see Dawson and Kouzarides 2012). In addition, chromatin remodelers have been implicated in cancer (discussed in Wilson and Roberts 2011). The first link between chromatin remodeling and cancer emanated from the discovery that SNF5 (*SMARCB1/BAF47*), a subunit of the human SWI/SNF remodeling complex, is inactivated in rhabdoid tumours (Biegel et al. 1999; Versteege et al. 1998). Homozygous *SNF5* deficiency results in embryonic lethality in mice whereas heterozygous loss predisposes to aggressive cancers, similar to those seen in humans (Guidi et al. 2001; Klochendler-Yeivin et al. 2000; Roberts et al. 2000). The mechanisms behind this have not been fully elucidated but given the central role for SWI/SNF in regulating nucleosome positioning it is not surprising that SWI/SNF components are mutated in 20 % of all human cancers (see Kim and Roberts 2014). Components of the SWI/SNF complex are also implicated in Coffin–Siris syndrome (Tsurusaki et al. 2012), again demonstrating that the outcome of particular mutations is context dependent.

Advances in high-throughput sequencing have also enabled mapping of mutations that are associated with cancer, which can help in identifying targets for diagnosis and treatment (Simo-Riudalbas and Esteller 2014). For example, pan-sequencing efforts have identified recurrent mutations in previously unknown chromatin genes such as *ASXL1* (Abdel-Wahab et al. 2012; Inoue et al. 2013), where mutations have been implicated in the loss of PRC2-mediated methylation of H3K27 leading to myeloid transformation. In addition, global DNA hypo-methylation and locus specific hyper-methylation in tumours allows DNA methylation signatures to be used as potential biomarkers in cancer diagnosis and prognosis (Fernandez et al. 2012). The promoter methylation level of the O^6^–methguanine-DNA methyltransferase (*MGMT*) gene, which encodes a DNA repair enzyme that protects cells against alkylating agents, has been shown to be a suitable marker for early detection of drug resistance. Hyper-methylation of the promoter correlates with good response of patients to alkylating antineoplastic drugs such as temozolomide (Wick et al. 2012). Changes in histone modification levels have also been used as prognostic markers. For example, lower levels of H3K4me2, H3K18ac and H3K9me2 are associated with poorer outcomes in lung and kidney cancer (Seligson et al. 2009). The ongoing improvements in epigenetic profiling techniques promise to rationalise the treatment of diseases in which epigenetic pathways are deregulated.

## Epigenetic therapies

The reversibility of epigenetic events makes the epigenetic machinery an attractive target for therapeutic intervention. A high number of drugs targeting epigenetic factors and/or pathways are currently being developed or tested in clinical trials (discussed in Heerboth et al. 2014). Amongst them are histone methyltransferase and de-methylase inhibitors (see McGrath and Trojer 2015) and BET inhibitors (reviewed in Barbieri et al. 2013), a class of drugs that compete for binding of BET proteins (bromodomain and extra terminal family proteins) to acetylated histones and cause the downregulation of c-myc in myc-dependent cancers. The majority of drugs currently approved for clinical use target DNA methylation and histone acetylation levels by inhibiting the DNMTs and HDACs (Table 2).

**Table 2:** Epigenetic drugs

Drug class	Drug	Target	Reference
DNA methylation inhibitors			
Nucleoside analogues	5-azacytidine (Vidaza)^a^	DNMT1	Cataldo et al. (2009)
	5-aza-2′-deoxycytidine (Decitabine)^a^	DNMT1	Saba (2007)
	Zebularine	DNMT1	Yoo et al. (2008)
	5-fluoro-2′-deoxycytidine	DNMT1	Beumer et al. (2008)
	S110	DNMT1	Chuang et al. (2010)
Antisense oligonucleotide	MG98	DNMT1	Amato et al. (2012)
Small molecule inhibitor	RG108	DNMT1	Graca et al. (2014)
HDAC inhibitors			
Hydroxamic acids	Suberoylanilide hydroxamic acid (Vorinostat)^a^	HDACs	Mann et al. (2007)
	Panabinostat^a^	HDACs	Richardson et al. (2015)
	Belinostat^a^	HDACs	Rashidi and Cashen (2015)
Depsipeptides	Romidepsin (Istodax)^a^	HDACs	Yazbeck and Grant (2015)
Short-chain fatty acids	Valproic acid (Depakene)^a^	HDACs	Phiel et al. (2001)
Benzamide derivatives	MS-275 (Entinostat)^a^	HDACs	See review by De Souza and Chatterji (2015)
	ITF-2357 (Givinostat)	HDACs	
	MGCD-0103 (Mocetinostat)	HDACs	
	PCI-24781 (Abexinostat)	HDACs	
Bromodomain inhibitors			
Small molecule inhibitors	JQ1	BET family proteins	Delmore et al. (2011); Filippakopoulos et al. (2010)
	I-BET151	BET family proteins	Dawson et al. (2011)
	I-BET726	BET family proteins	Wyce et al. (2013)
HAT inhibitors			
Small molecule inhibitor	C646	p300 (KAT3B)	Bowers et al. (2010)
Histone methyltransferase inhibitors			
Small molecule inhibitors	EPZ-5676	DOT1L (KMT4)	Daigle et al. (2013)
	EPZ-6438	EZH2 (KMT6)	Knutson et al. (2014)
	GSK126	EZH2 (KMT6)	McCabe et al. (2012)
	3-deazaneplanocin A (DZNep)	H3K27me3 and H4K20me3	Miranda et al. (2009)
	BIX-01294	EHMT1/2 (GLP/G9A; KMT1D/C)	Kubicek et al. (2007)
	UNC0642	EHMT1/2 (GLP/G9A; KMT1D/C)	Liu et al. (2013)
	A-366	EHMT1/2 (GLP/G9A; KMT1D/C)	Sweis et al. (2014)
Histone de-methylase inhibitors			
Tranylcypromines	ORY-1001	LSD1 (KDM1A)	See review by McGrath and Trojer (2015)
	GSK2879552	LSD1 (KDM1A)	

A list of drugs targeting chromatin regulators commonly associated with disease

Common synonyms for gene names are given in parentheses. For a more exhaustive overview of compounds targeting epigenetic regulators we refer the reader to several recent reviews (McGrath and Trojer 2015; Rodriguez-Paredes and Esteller 2011; Yang et al. 2010)

^a^Drugs approved for clinical use by the US Food and Drug Administration (FDA)

Nucleoside-mimicking drugs were shown to have anti-proliferative properties. 5-Azacytidine (Aza; commercially: Vidaza), a cytidine analogue in which nitrogen replaces the carbon 5 atom, was the first to be used in clinical applications. Aza intercalates into DNA during replication. DNMT1 recognises the nucleotide analogue and irreversibly links to the nitrogen resulting in DNMT1 degradation and therefore reduction of methylation in dividing cells (Kaminskas et al. 2005; Momparler 2005; Santi et al. 1984). More recently, less toxic and more stable analogues (e.g. Zebularine, 5-fluoro-2-deoxycytidine, S110) have been developed and are in clinical trials. Other DNMT1 inhibitors such as RG108 and the antisense oligonucleotide MG98 that blocks translation of the DNMT1 mRNA are also being tested as inhibitors of DNA methylation.

A second class of drugs that have been approved for clinical use are histone deacetylase inhibitors (HDACi). HDACs are linked to chromatin silencing and their inhibition is thought to promote the reactivation of inappropriately silenced genes such as tumour suppressors. HDAC inhibitors belong to different structural classes such as epoxyketones, benzamides and short chains fatty acids. Valproic acid (a fatty acid HDACi) functions as a competitive binder to acetyl groups on the N-terminal histone tails by blocking the catalytic site of HDACs. It is currently being used for treating mental disorders such as epilepsy and bipolar disorders and is in clinical trials for cancer (see Slingerland et al. 2014). SAHA, also known as Vorinostat, is an anti T cell lymphoma drug that acts as a chelator for zinc ions found in the active site of HDACs. It causes histone, p53 and Hsp90 hyper-acetylation leading to apoptosis of cancer cells and sensitisation of tumours to other antineoplastic drugs. SAHA is also being investigated as an anti-HIV drug (Archin et al. 2012). In addition to these clinically approved drugs, a large number of novel molecules targeting a variety of epigenetic regulators are currently under development or in clinical trials (Table 2) providing an exciting outlook for epigenetic therapies.

## Conclusions

The epigenetic regulation of chromatin affects many fundamental biological pathways, from basic DNA transactions such as transcription, replication and DNA repair, to the establishment and maintenance of cell identity during development and tissue homeostasis. Many diseases are caused by mutations in chromatin regulators. These are typically monogenic diseases with mutations inherited from the parents, acquired de novo in the parental germline or in somatic tissues, or complex polygenic diseases. Environmental insults resulting from malnutrition, stress or exposure to harmful chemicals can also have considerable impact on the epigenome. The realisation that deregulation of chromatin causes or contributes to the formation of diseases has sparked significant efforts to develop epigenetic therapies. Changes in gene expression resulting from aberrant epigenetic regulation are potentially reversible and drugs targeting the epigenetic machinery have proven effective in the treatment of various diseases. However, since chromatin regulators are expressed in many tissues and are critical for many basic biological processes, a disadvantage of epigenetic drugs to date is their broad specificity and potentially adverse side effects. The rapid developments in high throughput sequencing technologies are enabling the profiling of individual genomes and epigenomes. These signatures can be valuable biomarkers in disease diagnosis and prognosis, and to devise effective avenues for therapeutic intervention. The ongoing development of drugs with improved characteristics and genomic and epigenomic profiling promise to catalyse personalised treatment strategies that will allow effective use of epigenetic drugs alongside other drugs in combination therapies tailored to a patient’s condition.

## References

[CR1] Abdel-Wahab O (2012). ASXL1 mutations promote myeloid transformation through loss of PRC2-mediated gene repression. Cancer Cell.

[CR2] Alarcon JM, Malleret G, Touzani K, Vronskaya S, Ishii S, Kandel ER, Barco A (2004). Chromatin acetylation, memory, and LTP are impaired in CBP+/− mice: a model for the cognitive deficit in Rubinstein-Taybi syndrome and its amelioration. Neuron.

[CR3] Alisch RS, Wang T, Chopra P, Visootsak J, Conneely KN, Warren ST (2013). Genome-wide analysis validates aberrant methylation in fragile X syndrome is specific to the FMR1 locus. BMC Med Genet.

[CR4] Amato RJ, Stephenson J, Hotte S, Nemunaitis J, Belanger K, Reid G, Martell RE (2012). MG98, a second-generation DNMT1 inhibitor, in the treatment of advanced renal cell carcinoma. Cancer Investig.

[CR5] Amir RE, Van den Veyver IB, Wan M, Tran CQ, Francke U, Zoghbi HY (1999). Rett syndrome is caused by mutations in X-linked MECP2, encoding methyl-CpG-binding protein 2. Nat Genet.

[CR6] Anway MD, Leathers C, Skinner MK (2006). Endocrine disruptor vinclozolin induced epigenetic transgenerational adult-onset disease. Endocrinology.

[CR7] Archin NM (2012). Administration of vorinostat disrupts HIV-1 latency in patients on antiretroviral therapy. Nature.

[CR8] Balemans MC (2014). Reduced Euchromatin histone methyltransferase 1 causes developmental delay, hypotonia, and cranial abnormalities associated with increased bone gene expression in Kleefstra syndrome mice. Dev Biol.

[CR9] Bannister AJ, Kouzarides T (2011). Regulation of chromatin by histone modifications. Cell Res.

[CR10] Barbieri I, Cannizzaro E, Dawson MA (2013) Bromodomains as therapeutic targets in cancer. Brief Funct Genomics 12:219–230. doi:10.1093/bfgp/elt007 10.1093/bfgp/elt007 23543289

[CR11] Batsukh T (2010). CHD8 interacts with CHD7, a protein which is mutated in CHARGE syndrome. Hum Mol Genet.

[CR12] Behn-Krappa A, Doerfler W (1994). Enzymatic amplification of synthetic oligodeoxyribonucleotides: implications for triplet repeat expansions in the human genome. Hum Mutat.

[CR13] Berdasco M, Esteller M (2012). Hot topics in epigenetic mechanisms of aging: 2011. Aging Cell.

[CR14] Beumer JH, Parise RA, Newman EM, Doroshow JH, Synold TW, Lenz HJ, Egorin MJ (2008). Concentrations of the DNA methyltransferase inhibitor 5-fluoro-2'-deoxycytidine (FdCyd) and its cytotoxic metabolites in plasma of patients treated with FdCyd and tetrahydrouridine (THU). Cancer Chemother Pharmacol.

[CR15] Biegel JA, Zhou JY, Rorke LB, Stenstrom C, Wainwright LM, Fogelgren B (1999). Germ-line and acquired mutations of INI1 in atypical teratoid and rhabdoid tumors. Cancer Res.

[CR16] Bird A (2008). The methyl-CpG-binding protein MeCP2 and neurological disease. Biochem Soc Trans.

[CR17] Borgel J (2010). Targets and dynamics of promoter DNA methylation during early mouse development. Nat Genet.

[CR18] Bostick M, Kim JK, Esteve PO, Clark A, Pradhan S, Jacobsen SE (2007). UHRF1 plays a role in maintaining DNA methylation in mammalian cells. Science.

[CR19] Bouazoune K, Kingston RE (2012). Chromatin remodeling by the CHD7 protein is impaired by mutations that cause human developmental disorders. Proc Natl Acad Sci U S A.

[CR20] Bowers EM (2010). Virtual ligand screening of the p300/CBP histone acetyltransferase: identification of a selective small molecule inhibitor. Chem Biol.

[CR21] Cabianca DS, Casa V, Bodega B, Xynos A, Ginelli E, Tanaka Y, Gabellini D (2012). A long ncRNA links copy number variation to a polycomb/trithorax epigenetic switch in FSHD muscular dystrophy. Cell.

[CR22] Cantarino N, Douet J, Buschbeck M (2013). MacroH2A—an epigenetic regulator of cancer. Cancer Lett.

[CR23] Cardoso C, Timsit S, Villard L, Khrestchatisky M, Fontes M, Colleaux L (1998). Specific interaction between the XNP/ATR-X gene product and the SET domain of the human EZH2 protein. Hum Mol Genet.

[CR24] Cassidy SB, Schwartz S (1998). Prader-Willi and Angelman syndromes. Disord Genomic Imprinting Med.

[CR25] Cataldo VD, Cortes J, Quintas-Cardama A (2009). Azacitidine for the treatment of myelodysplastic syndrome. Expert Rev Anticancer Ther.

[CR26] Chaligne R, Heard E (2014). X-chromosome inactivation in development and cancer. FEBS Lett.

[CR27] Chen RZ, Akbarian S, Tudor M, Jaenisch R (2001). Deficiency of methyl-CpG binding protein-2 in CNS neurons results in a Rett-like phenotype in mice. Nat Genet.

[CR28] Chuang LS, Ian HI, Koh TW, Ng HH, Xu G, Li BF (1997). Human DNA-(cytosine-5) methyltransferase-PCNA complex as a target for p21WAF1. Science.

[CR29] Chuang JC (2010). S110, a 5-Aza-2'-deoxycytidine-containing dinucleotide, is an effective DNA methylation inhibitor in vivo and can reduce tumor growth. Mol Cancer Ther.

[CR30] Clayton-Smith J, Pembrey ME (1992). Angelman syndrome. J Med Genet.

[CR31] Cobb S, Guy J, Bird A (2010). Reversibility of functional deficits in experimental models of Rett syndrome. Biochem Soc Trans.

[CR32] Collins AL (2004). Mild overexpression of MeCP2 causes a progressive neurological disorder in mice. Hum Mol Genet.

[CR33] Daigle SR (2013). Potent inhibition of DOT1L as treatment of MLL-fusion leukemia. Blood.

[CR34] Dashwood RH, Ho E (2007). Dietary histone deacetylase inhibitors: from cells to mice to man. Semin Cancer Biol.

[CR35] Dawson MA, Kouzarides T (2012). Cancer epigenetics: from mechanism to therapy. Cell.

[CR36] Dawson MA (2011). Inhibition of BET recruitment to chromatin as an effective treatment for MLL-fusion leukaemia. Nature.

[CR37] De Souza C, Chatterji BP (2015) HDAC Inhibitors as Novel Anti-Cancer Therapeutics. Recent patents on anti-cancer drug discovery 10:145-162 10.2174/1574892810666150317144511 25782916

[CR38] Delhommeau F (2009). Mutation in TET2 in myeloid cancers. N Engl J Med.

[CR39] Delmore JE (2011). BET bromodomain inhibition as a therapeutic strategy to target c-Myc. Cell.

[CR40] Desplats P (2011). Alpha-synuclein sequesters Dnmt1 from the nucleus: a novel mechanism for epigenetic alterations in Lewy body diseases. The J Biol Chem.

[CR41] Eden A, Gaudet F, Waghmare A, Jaenisch R (2003). Chromosomal instability and tumors promoted by DNA hypomethylation. Science.

[CR42] Ehrlich M (2003). The ICF syndrome, a DNA methyltransferase 3B deficiency and immunodeficiency disease. Clin Immunol.

[CR43] Ehrlich M (2008). ICF, an immunodeficiency syndrome: DNA methyltransferase 3B involvement, chromosome anomalies, and gene dysregulation. Autoimmunity.

[CR44] Elliott HR (2014). Differences in smoking associated DNA methylation patterns in South Asians and Europeans. Clin Epigenetics.

[CR45] Ernst M (2005). Amygdala and nucleus accumbens in responses to receipt and omission of gains in adults and adolescents. NeuroImage.

[CR46] Feinberg AP, Vogelstein B (1983). Hypomethylation distinguishes genes of some human cancers from their normal counterparts. Nature.

[CR47] Fernandez AF (2012). A DNA methylation fingerprint of 1628 human samples. Genome Res.

[CR48] Filippakopoulos P (2010). Selective inhibition of BET bromodomains. Nature.

[CR49] Fraga MF (2005). Epigenetic differences arise during the lifetime of monozygotic twins. Proc Natl Acad Sci U S A.

[CR50] Francis D, Diorio J, Liu D, Meaney MJ (1999). Nongenomic transmission across generations of maternal behavior and stress responses in the rat. Science.

[CR51] Gershon A, Sudheimer K, Tirouvanziam R, Williams LM, O'Hara R (2013). The long-term impact of early adversity on late-life psychiatric disorders. Curr Psychiatry Rep.

[CR52] Gibbons RJ, Higgs DR (2000). Molecular-clinical spectrum of the ATR-X syndrome. Am J Med Genet.

[CR53] Gibbons RJ (1995). Clinical and hematologic aspects of the X-linked alpha-thalassemia/mental retardation syndrome (ATR-X). Am J Med Genet.

[CR54] Gibbons RJ, McDowell TL, Raman S, O'Rourke DM, Garrick D, Ayyub H, Higgs DR (2000). Mutations in ATRX, encoding a SWI/SNF-like protein, cause diverse changes in the pattern of DNA methylation. Nat Genet.

[CR55] Gibbons RJ (2008). Mutations in the chromatin-associated protein ATRX. Hum Mutat.

[CR56] Gibson WT (2012). Mutations in EZH2 cause Weaver syndrome. Am J Hum Genet.

[CR57] Gorbunova V, Seluanov A, Mittelman D, Wilson JH (2004). Genome-wide demethylation destabilizes CTG.CAG trinucleotide repeats in mammalian cells. Hum Mol Genet.

[CR58] Graca I (2014). Anti-tumoral effect of the non-nucleoside DNMT inhibitor RG108 in human prostate cancer cells. Curr Pharm Des.

[CR59] Guidi CJ (2001). Disruption of Ini1 leads to peri-implantation lethality and tumorigenesis in mice. Mol Cell Biol.

[CR60] Gurard-Levin ZA, Quivy JP, Almouzi G (2014). Histone chaperones: assisting histone traffic and nucleosome dynamics. Annu Rev Biochem.

[CR61] Guy J, Gan J, Selfridge J, Cobb S, Bird A (2007). Reversal of neurological defects in a mouse model of Rett syndrome. Science.

[CR62] Hansen RS, Wijmenga C, Luo P, Stanek AM, Canfield TK, Weemaes CM, Gartler SM (1999). The DNMT3B DNA methyltransferase gene is mutated in the ICF immunodeficiency syndrome. Proc Natl Acad Sci U S A.

[CR63] Hashimoto T (2005). Relationship of brain-derived neurotrophic factor and its receptor TrkB to altered inhibitory prefrontal circuitry in schizophrenia. The J Neurosci : the Off J Soc Neurosci.

[CR64] Hebbes TR, Clayton AL, Thorne AW, Crane-Robinson C (1994). Core histone hyperacetylation co-maps with generalized DNase I sensitivity in the chicken beta-globin chromosomal domain. The EMBO journal.

[CR65] Heerboth S, Lapinska K, Snyder N, Leary M, Rollinson S, Sarkar S (2014). Use of epigenetic drugs in disease: an overview. Genet Epigenetics.

[CR66] Hogart A, Nagarajan RP, Patzel KA, Yasui DH, Lasalle JM (2007). 15q11-13 GABAA receptor genes are normally biallelically expressed in brain yet are subject to epigenetic dysregulation in autism-spectrum disorders. Hum Mol Genet.

[CR67] Huh MS (2012). Compromised genomic integrity impedes muscle growth after Atrx inactivation. J Clin Invest.

[CR68] Inoue D (2013). Myelodysplastic syndromes are induced by histone methylation-altering ASXL1 mutations. J Clin Invest.

[CR69] Ishigaki S (2012). Position-dependent FUS-RNA interactions regulate alternative splicing events and transcriptions. Scientific Rep.

[CR70] Iwase S (2007). The X-linked mental retardation gene SMCX/JARID1C defines a family of histone H3 lysine 4 demethylases. Cell.

[CR71] Janssen C, Schmalbach S, Boeselt S, Sarlette A, Dengler R, Petri S (2010). Differential histone deacetylase mRNA expression patterns in amyotrophic lateral sclerosis. J Neuropathol Exp Neurol.

[CR72] Jensen LR (2005). Mutations in the JARID1C gene, which is involved in transcriptional regulation and chromatin remodeling, cause X-linked mental retardation. Am J Hum Genet.

[CR73] Jenuwein T, Allis CD (2001). Translating the histone code. Science.

[CR74] Jiang YL (2005). DNMT3B mutations and DNA methylation defect define two types of ICF syndrome. Hum Mutat.

[CR75] Jimenez-Chillaron JC, Diaz R, Martinez D, Pentinat T, Ramon-Krauel M, Ribo S, Plotch T (2012). The role of nutrition on epigenetic modifications and their implications on health. Biochimie.

[CR76] Jin P, Warren ST (2000). Understanding the molecular basis of fragile X syndrome. Hum Mol Genet.

[CR77] Jin B (2008). DNA methyltransferase 3B (DNMT3B) mutations in ICF syndrome lead to altered epigenetic modifications and aberrant expression of genes regulating development, neurogenesis and immune function. Hum Mol Genet.

[CR78] Johansson M, Rastam M, Billstedt E, Danielsson S, Stromland K, Miller M, Gillberg C (2006). Autism spectrum disorders and underlying brain pathology in CHARGE association. Dev Med Child Neurol.

[CR79] Jones PA, Laird PW (1999). Cancer epigenetics comes of age. Nat Genet.

[CR80] Jones PL (1998). Methylated DNA and MeCP2 recruit histone deacetylase to repress transcription. Nat Genet.

[CR81] Jowaed A, Schmitt I, Kaut O, Wuller U (2010). Methylation regulates alpha-synuclein expression and is decreased in Parkinson's disease patients' brains. The J Neurosci : the Off J Soc Neurosci.

[CR82] Jurkowska RZ, Jurkowski TP, Jeltch A (2011). Structure and function of mammalian DNA methyltransferases. Chembiochem: European Journal of Chemical Biology.

[CR83] Kaminskas E, Farrell AT, Wang YC, Sridhara R, Pazdur R (2005). FDA drug approval summary: azacitidine (5-azacytidine, Vidaza) for injectable suspension. Oncologist.

[CR84] Kim KH, Roberts CW (2014). Mechanisms by which SMARCB1 loss drives rhabdoid tumor growth. Cancer Genet.

[CR85] Kishino T, Lalande M, Wagstaff J (1997). UBE3A/E6-AP mutations cause Angelman syndrome. Nat Genet.

[CR86] Kleefstra T (2006). Loss-of-function mutations in euchromatin histone methyl transferase 1 (EHMT1) cause the 9q34 subtelomeric deletion syndrome. Am J Hum Genet.

[CR87] Kleine-Kohlbrecher D (2010). A functional link between the histone demethylase PHF8 and the transcription factor ZNF711 in X-linked mental retardation. Mol Cell.

[CR88] Klochendler-Yeivin A, Fiette L, Barra J, Muchardt C, Babinet C, Yaniv M (2000). The murine SNF5/INI1 chromatin remodeling factor is essential for embryonic development and tumor suppression. EMBO Rep.

[CR89] Knutson SK (2014). Selective inhibition of EZH2 by EPZ-6438 leads to potent antitumor activity in EZH2-mutant non-Hodgkin lymphoma. Mol Cancer Ther.

[CR90] Kramer JM, van Bokhoven H (2009). Genetic and epigenetic defects in mental retardation. Int J Biochem Cell Biol.

[CR91] Krumm N, O'Roak BJ, Shendure J, Eichler EE (2014). A de novo convergence of autism genetics and molecular neuroscience. Trends Neurosci.

[CR92] Kubicek S (2007). Reversal of H3K9me2 by a small-molecule inhibitor for the G9a histone methyltransferase. Mol Cell.

[CR93] Kurotaki N (2002). Haploinsufficiency of NSD1 causes Sotos syndrome. Nat Genet.

[CR94] Laumonnier F (2005). Mutations in PHF8 are associated with X linked mental retardation and cleft lip/cleft palate. J Med Genet.

[CR95] Laurent L (2010). Dynamic changes in the human methylome during differentiation. Genome Res.

[CR96] Law MJ (2010). ATR-X syndrome protein targets tandem repeats and influences allele-specific expression in a size-dependent manner. Cell.

[CR97] Lazo-Gomez R, Ramirez-Jarquin UN, Tovar YRLB, Tapia R (2013). Histone deacetylases and their role in motor neuron degeneration. Front Cell Neurosci.

[CR98] Lechner MS, Schultz DC, Negorev D, Maul GG, Rauscher FJ (2005). The mammalian heterochromatin protein 1 binds diverse nuclear proteins through a common motif that targets the chromoshadow domain. Biochem Biophys Res Commun.

[CR99] Lederer D (2012). Deletion of KDM6A, a histone demethylase interacting with MLL2, in three patients with Kabuki syndrome. Am J Hum Genet.

[CR100] Lee JV (2014). Akt-dependent metabolic reprogramming regulates tumor cell histone acetylation. Cell Metab.

[CR101] Lewis PW, Elsaesser SJ, Noh KM, Stadler SC, Allis CD (2010). Daxx is an H3.3-specific histone chaperone and cooperates with ATRX in replication-independent chromatin assembly at telomeres. Proc Natl Acad Sci U S A.

[CR102] Ley TJ (2010). DNMT3A mutations in acute myeloid leukemia. N Engl J Med.

[CR103] Li E, Zhang Y (2014). DNA methylation in mammals. Cold Spring Harb Perspect Biol.

[CR104] Lister R (2009). Human DNA methylomes at base resolution show widespread epigenomic differences. Nature.

[CR105] Liu F (2013). Discovery of an in vivo chemical probe of the lysine methyltransferases G9a and GLP. J Med Chem.

[CR106] Lu H, Liu X, Deng Y, Qing H (2013). DNA methylation, a hand behind neurodegenerative diseases. Front Aging Neurosci.

[CR107] Luger K, Dechassa ML, Tremethick DJ (2012). New insights into nucleosome and chromatin structure: an ordered state or a disordered affair?. Nat Rev Mol Cell Biol.

[CR108] Lyst MJ (2013). Rett syndrome mutations abolish the interaction of MeCP2 with the NCoR/SMRT co-repressor. Nat Neurosci.

[CR109] Maffini MV, Rubin BS, Sonnenschein C, Soto AM (2006). Endocrine disruptors and reproductive health: the case of bisphenol-A. Mol Cell Endocrinol.

[CR110] Mallery DL (1998). Molecular analysis of mutations in the CSB (ERCC6) gene in patients with Cockayne syndrome. Am J Hum Genet.

[CR111] Mann BS (2007). Vorinostat for treatment of cutaneous manifestations of advanced primary cutaneous T-cell lymphoma. Clin Cancer Res : an Off J Am Assoc Cancer Res.

[CR112] Matsumoto L, Takuma H, Tamaoka A, Kurisaki H, Date H, Tsuji S, Iwata A (2010). CpG demethylation enhances alpha-synuclein expression and affects the pathogenesis of Parkinson's disease. PLoS One.

[CR113] Matsuura T (1997). De novo truncating mutations in E6-AP ubiquitin-protein ligase gene (UBE3A) in Angelman syndrome. Nat Genet.

[CR114] McCabe MT (2012). EZH2 inhibition as a therapeutic strategy for lymphoma with EZH2-activating mutations. Nature.

[CR115] McDowell TL (1999). Localization of a putative transcriptional regulator (ATRX) at pericentromeric heterochromatin and the short arms of acrocentric chromosomes. Proc Natl Acad Sci U S A.

[CR116] McGrath J, Trojer P (2015). Targeting histone lysine methylation in cancer. Pharmacol Ther.

[CR117] Miranda TB (2009). DZNep is a global histone methylation inhibitor that reactivates developmental genes not silenced by DNA methylation. Mol Cancer Ther.

[CR118] Miyake N (2013). MLL2 and KDM6A mutations in patients with Kabuki syndrome. Am J Med Genet A.

[CR119] Momparler RL (2005). Pharmacology of 5-Aza-2'-deoxycytidine (decitabine). Semin Hematol.

[CR120] Nan X, Ng HH, Johnson CA, Laherty CD, Turner BM, Eisenman RN, Bird A (1998). Transcriptional repression by the methyl-CpG-binding protein MeCP2 involves a histone deacetylase complex. Nature.

[CR121] Narlikar GJ, Sundaramoorthy R, Owen-Hughes T (2013). Mechanisms and functions of ATP-dependent chromatin-remodeling enzymes. Cell.

[CR122] Ng SB (2010). Exome sequencing identifies MLL2 mutations as a cause of Kabuki syndrome. Nat Genet.

[CR123] Ogawa H, Ishiguro K, Gaubatz S, Livingston DM, Nakatani Y (2002). A complex with chromatin modifiers that occupies E2F- and Myc-responsive genes in G0 cells. Science.

[CR124] Park JH, Stoffers DA, Nicholls RD, Simmons RA (2008). Development of type 2 diabetes following intrauterine growth retardation in rats is associated with progressive epigenetic silencing of Pdx1. J Clin Invest.

[CR125] Pazin MJ, Kamakaka RT, Kadonaga JT (1994). ATP-dependent nucleosome reconfiguration and transcriptional activation from preassembled chromatin templates. Science.

[CR126] Perroud N (2013). Response to psychotherapy in borderline personality disorder and methylation status of the BDNF gene. Translational psychiatry.

[CR127] Petrij F (1995). Rubinstein-Taybi syndrome caused by mutations in the transcriptional co-activator CBP. Nature.

[CR128] Phiel CJ, Zhang F, Huang EY, Guenther MG, Lazar MA, Klein PS (2001). Histone deacetylase is a direct target of valproic acid, a potent anticonvulsant, mood stabilizer, and teratogen. The J Biol Chem.

[CR129] Pieper HC, Evert BO, Kaut O, Riederer PF, Waha A, Wuller U (2008). Different methylation of the TNF-alpha promoter in cortex and substantia nigra: Implications for selective neuronal vulnerability. Neurobiol Dis.

[CR130] Poirier LA, Vlasova TI (2002). The prospective role of abnormal methyl metabolism in cadmium toxicity. Environ Health Perspect.

[CR131] Pombo A, Dillon N (2015). Three-dimensional genome architecture: players and mechanisms. Nat Rev Mol Cell Biol.

[CR132] Prokocimer M, Barkan R, Gruenbaum Y (2013). Hutchinson-Gilford progeria syndrome through the lens of transcription. Aging Cell.

[CR133] Radford EJ (2014). In utero effects. In utero undernourishment perturbs the adult sperm methylome and intergenerational metabolism. Science.

[CR134] Rashidi A, Cashen AF (2015). Belinostat for the treatment of relapsed or refractory peripheral T-cell lymphoma. Future Oncol.

[CR135] Rayasam GV (2003). NSD1 is essential for early post-implantation development and has a catalytically active SET domain. The EMBO J.

[CR136] Richardson PG et al. (2015) Panobinostat: a novel pan-deacetylase inhibitor for the treatment of relapsed or relapsed and refractory multiple myeloma. Expert review of anticancer therapy:1-12 doi:10.1586/14737140.2015.1047770 10.1586/14737140.2015.1047770 26051506

[CR137] Roadmap Epigenomics C (2015). Integrative analysis of 111 reference human epigenomes. Nature.

[CR138] Roberts CW, Galusha SA, McMenamin ME, Fletcher CD, Orkin SH (2000). Haploinsufficiency of Snf5 (integrase interactor 1) predisposes to malignant rhabdoid tumors in mice. Proc Natl Acad Sci U S A.

[CR139] Rodriguez-Paredes M, Esteller M (2011). Cancer epigenetics reaches mainstream oncology. Nat Med.

[CR140] Roelfsema JH (2005). Genetic heterogeneity in Rubinstein-Taybi syndrome: mutations in both the CBP and EP300 genes cause disease. Am J Hum Genet.

[CR141] Ronn T (2008). Age influences DNA methylation and gene expression of COX7A1 in human skeletal muscle. Diabetologia.

[CR142] Roopra A, Qazi R, Schoenike B, Daley TJ, Morrison JF (2004). Localized domains of G9a-mediated histone methylation are required for silencing of neuronal genes. Mol Cell.

[CR143] Roth TL, Lubin FD, Funk AJ, Sweatt JD (2009). Lasting epigenetic influence of early-life adversity on the BDNF gene. Biol Psychiatry.

[CR144] Saba HI (2007). Decitabine in the treatment of myelodysplastic syndromes. Ther Clin Risk Manag.

[CR145] Salnikow K, Costa M (2000). Epigenetic mechanisms of nickel carcinogenesis. Journal of Environmental Pathology, Toxicology and Oncology: official organ of the International Society for Environmental Toxicology and Cancer.

[CR146] Samaco RC, Hogart A, LaSalle JM (2005). Epigenetic overlap in autism-spectrum neurodevelopmental disorders: MECP2 deficiency causes reduced expression of UBE3A and GABRB3. Hum Mol Genet.

[CR147] Sandovici I (2011). Maternal diet and aging alter the epigenetic control of a promoter-enhancer interaction at the Hnf4a gene in rat pancreatic islets. Proc Natl Acad Sci U S A.

[CR148] Sanli I, Feil R (2015). Chromatin mechanisms in the developmental control of imprinted gene expression. Int J Biochem Cell Biol.

[CR149] Santi DV, Norment A, Garrett CE (1984). Covalent bond formation between a DNA-cytosine methyltransferase and DNA containing 5-azacytosine. Proc Natl Acad Sci U S A.

[CR150] Sarma K (2014). ATRX directs binding of PRC2 to Xist RNA and Polycomb targets. Cell.

[CR151] Schaefer A (2009). Control of cognition and adaptive behavior by the GLP/G9a epigenetic suppressor complex. Neuron.

[CR152] Schnetz MP (2009). Genomic distribution of CHD7 on chromatin tracks H3K4 methylation patterns. Genome Res.

[CR153] Schwartz JC, Ebmeier CC, Podell ER, Heimiller J, Taatjes DJ, Cech TR (2012). FUS binds the CTD of RNA polymerase II and regulates its phosphorylation at Ser2. Genes Dev.

[CR154] Seligson DB (2009). Global levels of histone modifications predict prognosis in different cancers. The Am J Pathol.

[CR155] Sharif J (2007). The SRA protein Np95 mediates epigenetic inheritance by recruiting Dnmt1 to methylated DNA. Nature.

[CR156] Shen L (2007). Genome-wide profiling of DNA methylation reveals a class of normally methylated CpG island promoters. PLoS Genet.

[CR157] Shenker NS (2013). Epigenome-wide association study in the European Prospective Investigation into Cancer and Nutrition (EPIC-Turin) identifies novel genetic loci associated with smoking. Hum Mol Genet.

[CR158] Shiao YH, Crawford EB, Anderson LM, Patel P, Ko K (2005). Allele-specific germ cell epimutation in the spacer promoter of the 45S ribosomal RNA gene after Cr(III) exposure. Toxicol Appl Pharmacol.

[CR159] Shogren-Knaak M, Ishii H, Sun JM, Pazin MJ, Davie JR, Peterson CL (2006). Histone H4-K16 acetylation controls chromatin structure and protein interactions. Science.

[CR160] Simo-Riudalbas L, Esteller M (2014). Cancer genomics identifies disrupted epigenetic genes. Hum Genet.

[CR161] Slingerland M, Guchelaar HJ, Gelderblom H (2014). Histone deacetylase inhibitors: an overview of the clinical studies in solid tumors. Anti-Cancer Drugs.

[CR162] Strahl BD, Allis CD (2000). The language of covalent histone modifications. Nature.

[CR163] Sutcliffe JS, Nelson DL, Zhang F, Pieretti M, Caskey CT, Saxe D, Warren ST (1992). DNA methylation represses FMR-1 transcription in fragile X syndrome. Hum Mol Genet.

[CR164] Sweis RF (2014). Discovery and development of potent and selective inhibitors of histone methyltransferase g9a. ACS Med Chem Lett.

[CR165] Tahiliani M (2007). The histone H3K4 demethylase SMCX links REST target genes to X-linked mental retardation. Nature.

[CR166] Takahashi H, McCaffery JM, Irizarry RA, Boeke JD (2006). Nucleocytosolic acetyl-coenzyme a synthetase is required for histone acetylation and global transcription. Mol Cell.

[CR167] Tatton-Brown K (2011). Germline mutations in the oncogene EZH2 cause Weaver syndrome and increased human height. Oncotarget.

[CR168] Tatton-Brown K (2013). Weaver syndrome and EZH2 mutations: clarifying the clinical phenotype. Am J Med Genet A.

[CR169] Tohgi H, Utsugisawa K, Nagane Y, Yoshimura M, Genda Y, Ukitsu M (1999). Reduction with age in methylcytosine in the promoter region –224 approximately –101 of the amyloid precursor protein gene in autopsy human cortex. Brain Research Mol Brain Res.

[CR170] Tsurusaki Y (2012). Mutations affecting components of the SWI/SNF complex cause Coffin-Siris syndrome. Nat Genet.

[CR171] Vardabasso C, Hasson D, Ratnakumar K, Chung CY, Duarte LF, Bernstein E (2014). Histone variants: emerging players in cancer biology. Cellular Mol Life Sci: CMLS.

[CR172] Versteege I (1998). Truncating mutations of hSNF5/INI1 in aggressive paediatric cancer. Nature.

[CR173] Veurink M, Koster M, Berg LT (2005) The history of DES, lessons to be learned. Pharmacy World & Science 27:139-143 doi:10.1007/s11096-005-3663-z 10.1007/s11096-005-3663-z 16096877

[CR174] Vissers LE (2004). Mutations in a new member of the chromodomain gene family cause CHARGE syndrome. Nat Genet.

[CR175] Wang YH, Griffith J (1996). Methylation of expanded CCG triplet repeat DNA from fragile X syndrome patients enhances nucleosome exclusion. The J Biol Chem.

[CR176] Wang X (2008). Induced ncRNAs allosterically modify RNA-binding proteins in cis to inhibit transcription. Nature.

[CR177] Wang J, Tang J, Lai M, Zhang H (2014). 5-Hydroxymethylcytosine and disease. Mutat Res Rev Mutat Res.

[CR178] Waterland RA (2006). Assessing the effects of high methionine intake on DNA methylation. The J Nutri.

[CR179] Waterland RA (2010). Season of conception in rural gambia affects DNA methylation at putative human metastable epialleles. PLoS Genet.

[CR180] Weaver IC, Meaney MJ, Szyf M (2006). Maternal care effects on the hippocampal transcriptome and anxiety-mediated behaviors in the offspring that are reversible in adulthood. Proc Natl Acad Sci U S A.

[CR181] Wellen KE, Hatzivassiliou G, Sachdeva UM, Bui TV, Cross JR, Thompson CB (2009). ATP-citrate lyase links cellular metabolism to histone acetylation. Science.

[CR182] Wick W (2012). Temozolomide chemotherapy alone versus radiotherapy alone for malignant astrocytoma in the elderly: the NOA-08 randomised, phase 3 trial. The Lancet Oncol.

[CR183] Williams SR (2010). Haploinsufficiency of HDAC4 causes brachydactyly mental retardation syndrome, with brachydactyly type E, developmental delays, and behavioral problems. Am J Hum Genet.

[CR184] Wilson VL, Jones PA (1983). DNA methylation decreases in aging but not in immortal cells. Science.

[CR185] Wilson BG, Roberts BG (2011). SWI/SNF nucleosome remodellers and cancer. Nat Rev Cancer.

[CR186] Wyce A (2013). BET inhibition silences expression of MYCN and BCL2 and induces cytotoxicity in neuroblastoma tumor models. PLoS One.

[CR187] Xie ZH (2006). Mutations in DNA methyltransferase DNMT3B in ICF syndrome affect its regulation by DNMT3L. Hum Mol Genet.

[CR188] Xu GL (1999). Chromosome instability and immunodeficiency syndrome caused by mutations in a DNA methyltransferase gene. Nature.

[CR189] Xue Y (2003). The ATRX syndrome protein forms a chromatin-remodeling complex with Daxx and localizes in promyelocytic leukemia nuclear bodies. Proc Natl Acad Sci U S A.

[CR190] Yang X, Lay F, Han H, Jones PA (2010). Targeting DNA methylation for epigenetic therapy. Trends Pharmacol Sci.

[CR191] Yazbeck VY, Grant S (2015) Romidepsin for the treatment of non-Hodgkin's lymphoma. Expert Opinion on Investigational Drugs:1–15. doi:10.1517/13543784.2015.1041586 10.1517/13543784.2015.1041586 25936363

[CR192] Yoo CB (2008). Long-term epigenetic therapy with oral zebularine has minimal side effects and prevents intestinal tumors in mice. Cancer Prev Res.

[CR193] Yun M, Wu J, Workman JL, Li B (2011). Readers of histone modifications. Cell Res.

[CR194] Zaidi S (2013). De novo mutations in histone-modifying genes in congenital heart disease. Nature.

[CR195] Zampieri M, Ciccarone F, Calabrese R, Franceschi C, Burkle A, Caiafa P (2015). Reconfiguration of DNA methylation in aging. Mech Ageing Dev.

[CR196] Zentner GE, Layman WS, Martin DM, Scacheri PC (2010). Molecular and phenotypic aspects of CHD7 mutation in CHARGE syndrome. Am J Med Genet A.

[CR197] Zochbauer-Muller S, Minna JD, Gazdar AF (2002). Aberrant DNA methylation in lung cancer: biological and clinical implications. Oncologist.

